# Akhirin Preserves Hemostatic Wound Repair Through Non-Hematopoietic Regulation of the Vascular Injury Microenvironment

**DOI:** 10.3390/jdb14030041

**Published:** 2026-09-07

**Authors:** Mohammad Badrul Anam, Mikiko Kudo, Terumasa Umemoto, Keisuke Yamashita, Rie Kawano, Kunimasa Ohta

**Affiliations:** 1 Department of Histology, Graduate School of Medical Sciences, Kumamoto University, Kumamoto 860-8556, Japan; anamm@upstate.edu; 2 Department of Neuroscience and Physiology, SUNY Upstate Medical University, Syracuse, NY 13210, USA; 3 Department of Stem Cell Biology, Graduate School of System Life Sciences, Kyushu University, Fukuoka 819-0395, Japan; k.mikiko@uq.edu.au (M.K.); yamashita@artsci.kyushu-u.ac.jp (K.Y.); 4 School of Biomedical Sciences, Faculty of Medicine, The University of Queensland, Brisbane, QLD 4072, Australia; 5 International Research Center for Medical Sciences, Kumamoto University, Kumamoto 860-0811, Japan; umemoto@kumamoto-u.ac.jp; 6 Department of Stem Cell Biology, Faculty of Arts and Science, Kyushu University, Fukuoka 819-0395, Japan; 7 Department of Medical Oncology and Hematology, Oita University Faculty of Medicine, Oita 879-5593, Japan; riekawa@oita-u.ac.jp

**Keywords:** Akhirin, hemostasis, wound healing, vascular injury, fibrinolysis, plasminogen activator system, non-hematopoietic regulation

## Abstract

Hemostasis and wound healing are highly coordinated processes that involve rapid clot formation followed by controlled remodeling of the injured tissue microenvironment. Prior work on Akhirin (AKH), a secreted extracellular matrix protein containing two von Willebrand factor A domains and an LCCL domain, has established it as a regulator of the neural stem niche in the developing brain and spinal cord injury microenvironment. However, its role as a non-hematopoietic molecule in the vascular injury response remains unknown. Here, we present evidence that AKH contributes to hemostasis and wound repair outside the neural niche. Our immunohistochemical and biochemical analyses demonstrated AKH around arterial tissues, suggesting a potential role at the blood-vessel interface. AKH-deficient mice exhibited a striking phenotype characterized by prolonged tail bleeding and delayed wound closure, indicating impaired vascular injury repair in vivo. Furthermore, bone marrow transplantation failed to rescue the prolonged bleeding phenotype, supporting a predominant non-hematopoietic contribution. Intriguingly, analysis of classical coagulation revealed an apparent paradox: activated partial thromboplastin time was shortened, whereas prothrombin time was not significantly altered. In contrast, increased expression of tissue plasminogen activator, urokinase-type plasminogen activator, and urokinase-type plasminogen activator receptor in AKH-deficient samples suggested dysregulated local fibrinolytic remodeling. Together, these findings identify AKH as a previously unrecognized extracellular regulator of hemostatic wound repair. Rather than indicating a defect in classical coagulation cascade activation, the findings associate AKH deficiency with impaired hemostatic control and altered expression of plasminogen activator system components, suggesting a role for AKH in the local vascular injury response.

## 1. Introduction

The mechanisms underlying hemostasis have been extensively studied because of their clinical significance in bleeding and thrombotic disorders, as well as their translational scope for therapeutic development. The intricate biological process of hemostasis comprises vascular constriction, platelet activation and aggregation, and fibrin polymerization events, which together prevent blood loss shortly after tissue injury [1,2]. Moreover, the barrier produced after the injury facilitates wound healing and comprises a transient extracellular matrix (ECM) that recruits and supports inflammatory cells, stromal activation, angiogenesis, re-epithelialization, and subsequent tissue remodeling [3,4,5]. Therefore, hemostasis and wound healing are highly regulated paired processes that require temporal and spatial harmonization of a variety of cell types and their secreted molecules [6]. The biological success of wound repair thus depends on a stable hemostatic clot formation and subsequent controlled remodeling of the fibrin scaffold within the injured tissue microenvironment [4,5].

Bleeding abnormalities often co-exist with defective repair systems; for example fibrinogen deficiency not only impairs hemostatic competence but also highlights the importance of an intact fibrin scaffold during tissue remodeling [7]. In addition, plasminogen activator (tPA), urokinase-type plasminogen activator (uPA), and urokinase-type plasminogen activator receptor (uPAR) constitute the plasminogen activator system, which has important roles beyond fibrinolysis [8,9]. This system contributes to the tight regulation of ECM turnover, immune cell migration, angiogenesis and tissue repair [10,11]. Both persistent fibrin accumulation and excessive fibrinolysis can be detrimental: persistent fibrin may obstruct normal remodeling, whereas excessive fibrinolysis can destabilize the hemostatic plug and disrupt the transient matrix required for effective repair [9,10,12]. An instructive example is plasminogen activator inhibitor-1 deficiency in humans, which is associated with bleeding abnormalities and prolonged wound healing [13,14]. These observations support a key principle of vascular injury biology: molecular cues that regulate clot stability and fibrinolytic balance can influence both resistance to hemorrhages and tissue repair [8,10,13]. Some of these cues have non-hematopoietic origins and can significantly influence whether a tissue injury response remains localized, stabilized and appropriately remodeled [5]. Accordingly, routine plasma-based coagulation assays may not fully explain abnormal bleeding phenotypes observed in vivo [2,14]. In such scenarios, defects may involve hemostatic plug maintenance, clot architecture, or the transition from hemostasis to wound healing rather than clot initiation alone [11,12]. Therefore, identifying ECM-derived and non-hematopoietic molecular cues that regulate the local injury niche remains an important challenge in hemostasis and wound-healing research.

AKH was originally discovered as a soluble ECM protein expressed in chicken eye [15]. Its molecular structure includes two von Willebrand factor A domains and one LCCL domain, suggesting potential roles in processes involving adhesion, proteolytic remodeling and matrix stability [16]. Subsequent studies have mainly characterized AKH as a neurodevelopmental regulator of neural stem/progenitor niches in the spinal cord and neurogenic regions of postnatal mouse brain [17,18]. Recent work also implicates AKH in innate immune barrier function through domain-specific processing and bacterial binding [19]. Given that vascular injury and repair similarly require coordinated ECM remodeling, cell adhesion, immune regulation, and matrix stabilization, these features raise the possibility that AKH may also contribute to vascular biology. Following vascular injury, a tightly regulated sequence of biological events occurs at the injury site, including adhesive interactions, proteolytic balance, immune regulation and matrix stabilization [12,20]. Accordingly, investigating the contribution of AKH to vascular niches, particularly during peripheral injury responses, may reveal a previously unidentified role in hemostasis and wound repair.

Here, we demonstrate that AKH, a previously unrecognized non-hematopoietic molecular cue, contributes to hemostasis and wound healing. Our immunohistochemical and Western blot findings support an association between AKH and the vascular environment, including enrichment around arterial structures. Most importantly, AKH deficiency produces a substantial bleeding phenotype in vivo that is normalized by bone marrow transplantation and is associated with delayed wound repair. However, the bleeding phenotype cannot readily be explained by the impairment of the classical coagulation cascade, as the prothrombin time (PT) and activated partial thromboplastin time (APTT) results show an apparent paradox. Instead, increased expression of tPA, uPA, and uPAR points to dysregulation of the local vascular injury response.

Overall, these findings position AKH as a non-hematopoietic, ECM-associated cue at the intersection of hemostasis, fibrinolytic regulation and wound healing, extending the known biological framework of AKH beyond the neural niche.

## 2. Material and Methods

### 2.1. Animal

All experiments on mice were performed in accordance with the guidelines of the Committee on Animal Research at Kumamoto University and Kyushu University. The generation of AKH knockout mice has been described previously [17,18,19]. All mice were maintained on a mixed C57BL/6 strain background, and mice of both sexes were used.

### 2.2. Tail Bleeding Assay

Mice approximately 2 months of age were weighed before anesthesia and then deeply anesthetized. Animals were positioned prone and a 1.5 cm segment of the distal tail was removed with sterile scissors. Tails were immediately immersed 2 cm below the surface of pre-warmed (37 °C) 1× phosphate-buffered saline (PBS) in a 50 mL falcon tube and maintained vertically throughout the assay. Bleeding and re-bleeding events were observed for 20 min in all animals, including those in which bleeding initially stopped. Bleeding time was recorded using a stopwatch. For animals showing intermittent bleeding, the cumulative duration of all bleeding episodes during the 20 min period was used as the bleeding time. The assay was terminated at 20 min sharp to minimize mortality, as required by the institutional animal ethics protocol. Following assay termination, each mouse was weighed again together with the excised tail segment. Estimated blood loss was calculated from the reduction in body weight between the pre and post assay measurements using a gravimetric approach for murine tail bleeding assays; for volume estimation, 1 g of weight loss was considered approximately equivalent to 1 mL of blood [21].

### 2.3. Bone Marrow Transplantation Assay

Recipient mice were approximately 2 months old at the start of the bone marrow transplantation assay. Both AKH+/+ and AKH-/- recipient mice were conditioned with lethal total-body irradiation at a total dose of 10 Gy, delivered as two 5 Gy doses separated by 3 h. Within 24 h after irradiation, recipient mice received 1 × 10^7^ WT whole bone marrow cells suspended in 200 µL DMEM by intravenous tail vein injection. Whole bone marrow cells were isolated from femurs and tibias, filtered through a 70 µm cell strainer, washed, counted and resuspended immediately before transplantation. After transplantation, recipient mice were maintained under standard housing conditions for up to 3 months to recover, after which hemostatic function was evaluated using the tail bleeding assay described above. This experiment was performed to determine whether restoration of the hematopoietic compartment was sufficient to rescue the AKH-deficient bleeding phenotype.

### 2.4. Blood Collection and Plasma Preparation

Blood samples were collected from mice via tail bleeding or cardiac puncture under isoflurane anesthesia using a 23-G needle. For cardiac puncture, syringes were pre-coated with EDTA to prevent coagulation by chelating calcium ions (Factor IV). For tail bleeding, blood was collected directly into microcentrifuge tubes containing EDTA. Collected blood samples were centrifuged at 1500× *g* for 10 min at 4 °C, and the plasma fraction was carefully collected for subsequent analysis.

### 2.5. Umbilical Cord Vessel Processing for Histology

Umbilical cord tissue was used as a vessel-rich preparation to assess the vascular/perivascular localization of AKH. Mice were deeply anesthetized and perfused transcranial with PBS and 4% paraformaldehyde (PFA) in PBS to fix the tissues. Umbilical cord vessels were fixed in 4% PFA overnight at 4 °C and then immersed in 30% sucrose for cryoprotection. Samples were embedded in OCT compound (Tissue-Tek^®^; Sakura Finetek, Torrance, CA, USA), frozen, and sectioned at 30 µm thickness using a cryostat (Leica Microsystems, Wetzlar, Germany) for subsequent histological analyses.

### 2.6. Immunohistochemistry

Cryosections were rehydrated in PBS, then permeabilized and blocked with 5% goat serum in PBS with 0.3% TritonX-100. Following overnight incubation with primary antibodies at 4 °C, sections were washed, incubated with secondary antibodies at room temperature (RT) for 2 h, and counterstained with Hoechst33342 (Invitrogen Molecular Probes, Eugene, OR, USA) before being mounted with 90% glycerol containing 2% propyl gallate for observation under the microscope. The primary antibodies used were as follows: Anti-AKH-C’ (1:1000, custom-generated antibody) and Anti-CD31 (1:200, Abcam ab256569, Cambridge, UK). The secondary antibodies used were as follows: goat anti-rat IgG conjugated to Alexa Fluor 488 (1:1000; Jackson ImmunoResearch, West Grove, PA, USA) and goat anti-rabbit IgG conjugated to Alexa Fluor 647 (1:1000; Jackson ImmunoResearch, West Grove, PA, USA). Images were acquired using a Leica TCS SP8 STED confocal microscope (Leica Biosystem, Wetzlar, Germany).

### 2.7. Western Blotting

Plasma was collected from 2-month-old mice and mixed immediately with 2× SDS sample buffer (250 mM Tris-HCl pH 6.8, 8% SDS, 40% glycerol, 0.02% bromophenol blue, 10% β-mercaptoethanol) and heated for 5 min at 95 °C. Samples were separated by SDS-PAGE using 7.14% polyacrylamide gels and transferred to PVDF membranes (Millipore, Tokyo, Japan) using a semi-dry transfer system (Bio-Rad, Hercules, CA, USA). Membranes were blocked in 5% non-fat dry milk (or 5% BSA, depending on antibody) in 0.1% TBS-T buffer (20 mM Tris-HCl pH 7.6, 150 mM NaCl, 0.1% Tween-20) for 30 min at RT. Membranes were then incubated overnight at 4 °C with primary antibodies against AKH (1:1000; custom-generated antibody) and GAPDH (1:1000; MBL life science, Tokyo, Japan) diluted in blocking buffer. After washing with 0.1% TBS-T, membranes were incubated with HRP-conjugated secondary antibodies for 1 h at RT. Signal was detected using an enhanced chemiluminescence substrate, and images were captured using a chemiluminescence imaging system (Image Quant LAS 4000, Global Life Sciences Technologies, Tokyo, Japan). Band intensities were quantified using ImageJ (version 2.9.0; National Institutes of Health, Bethesda, MD, USA) and normalized to GAPDH.

### 2.8. Coagulation Assays

PT and APTT were measured using Thrombocheck PT and Thrombocheck APTT(S) kits, respectively, according to the manufacturer’s instructions (Sysmex, Hyogo, Japan) with minor modifications. Blood samples were collected from AKH+/+ and AKH-/- animals into tubes containing 10 µL of 5 M EDTA as an anticoagulant. Plasma was separated by centrifugation and used immediately for coagulation assays without storage. For the PT assay, 100 µL plasma samples were incubated at 37 °C for 3 min, and clotting was initiated by the addition of pre-warmed PT reagent containing thromboplastin and calcium. The time required for clot formation was recorded in seconds. For the APTT assay, plasma samples were mixed with APTT reagent and incubated at 37 °C for 2 min. Coagulation was then initiated by adding 100 µL of pre-warmed calcium chloride solution, and the clotting time was recorded in seconds. All assays were performed in duplicate, and the mean value of the two measurements was used for subsequent analysis.

### 2.9. RNA Extraction, qRT-PCR

Total RNA was extracted from plasma samples using the RNeasy^®^ Mini Kit (QIAGEN, Cat No. 74106, Carlsbad, CA, USA), according to the manufacturer’s instructions. RNA concentration and purity were assessed spectrophotometrically by measuring the A260/A280 ratio, and 1 µg of total RNA was reverse-transcribed into cDNA using the SuperScript™ II Reverse Transcriptase kit (Invitrogen, Cat. No. 18064022, Carlsbad, CA, USA). Quantitative PCR analysis was performed using Luna^®^ universal qPCR master mix (New England BioLabs, Cat. No. M3003E, Tokyo, Japan) on a StepOneplus Real-Time PCR System (Applied Biosystems, Tokyo, Japan). The thermal cycling protocol consisted of 95 °C for 1 min, followed by 40 cycles of 95 °C for 15 s and 60 °C for 30 s. Relative gene expression levels were calculated using the ΔΔCt method and normalized to GAPDH or an appropriate endogenous control. The following primer sequences were used:

tPA, forward 5′-AGAGTGTGAGCTTTCTGGCT-3′ and reverse 5′-TGGACGGATACAGTCTGACG-3′;

uPA, forward 5′-CTCCAACATTCACTGGTGCAACT-3′ and reverse 5′-CATCAGATCTGTGGGCATGGT-3′;

uPAR, forward 5′-AACAGTGCCTGGATGTGGTG-3′ and reverse 5′-GAAGTGGAAGGTGTCGTTGTTG-3′;

PAI-1, forward 5′-AATGACTGGGTGAAGACACACACA-3′ and reverse 5′-TTCCACTGGCCGTTGAAGTAGA-3′;

PAI-3, forward 5′-GCAAACGAAGGGCAAGATT-3′ and reverse 5′-TCCTCAGCGTTTTCTCACTCA-3′;

MMP, forward 5′-AGCAAGGACCTCGTTTTCATT-3′ and reverse 5′-GTCAATCCCTGGAAAGTCTTCA-3′;

GAPDH, forward 5′-CAACTCCCTCAAGATTGTCAGCAA-3′ and reverse 5′-GGCATGGACTGTGGTCATGA-3′.

### 2.10. Wound-Healing Assay

Wound-healing assays were performed using 3-month-old AKH+/+ and AKH-/- mice. Under anesthesia, the dorsal hair was shaved to expose the skin. A circular area with a diameter of 1 cm was marked on the dorsal skin, and the skin was carefully excised along the marked circle using surgical scissors without damaging the underlying muscle. Wound closure was monitored on days 1, 3, 7, and 15 after injury. At each time point, mice were re-anesthetized, and the wound site was photographed together with a ruler for scale calibration. Wound areas were quantified from the photographs using ImageJ software (version 2.9.0; National Institutes of Health, Bethesda, MD, USA).

## 3. Result

### 3.1. AKH Is Associated with Vascular Structures and Is Present in Circulation

**Figure 1:**
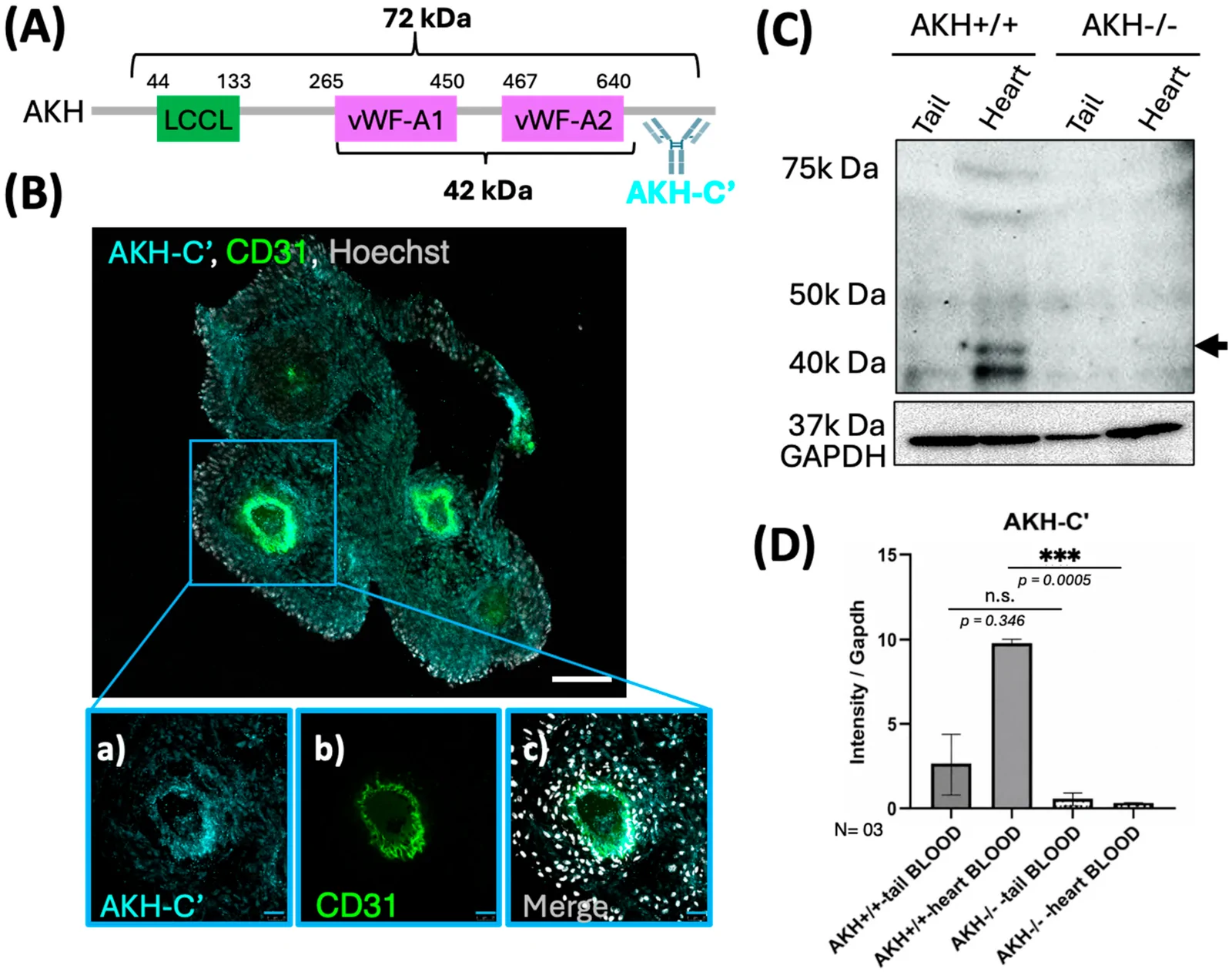
Detection of AKH in plasma and its localization around blood vessels in peripheral tissue. (**A**) A schematic illustration of the AKH protein structure. (**B**) Representative immunofluorescence images of peripheral tissue stained for AKH (cyan), CD31 (green) and Hoechst (grey). AKH signal was observed around CD31-positive vascular structures. Bar: 100 µm. (**a**–**c**) The boxed regions are shown at higher magnification on the right. Bars: 25 µm. (**C**) Western blot analysis of plasma from AKH+/+ and AKH-/- animals collected from the tail and heart. AKH-whole length (72 kDa) AKH-vWF domain fraction (~42 kDa) was detected in AKH+/+ plasma but was absent in AKH-/- samples. (**D**) Quantification of AKH-associated protein band intensity normalized by GAPDH. For panel D, one-way ANOVA was used. Data are shown as mean ± SEM; n.s., not significant; *** *p* < 0.001.

We investigated the spatial distribution of AKH in vascular tissue by immunofluorescence staining. For this analysis, we generated an antibody that recognizes the C-terminal region of AKH (AKH-C′; Figure 1A) and used it to detect AKH immunoreactivity. AKH immunoreactivity was observed around CD31-positive vascular structures, suggesting that AKH is enriched in the vascular microenvironment. Merged images showed an AKH signal surrounding CD31-positive endothelial structures, indicating that AKH may be associated with blood vessels and perivascular regions (Figure 1B).

To examine whether AKH protein is present in circulating blood components, we performed Western blot analysis using plasma samples collected from the tail and heart of 2-month-old control AKH+/+ mice and AKH-/- littermates. The predicted molecular weight of full-length AKH is approximately 72 kDa; however, only a weak band at this expected size was observed in AKH+/+ heart-derived plasma. Instead, under the same exposure conditions, a stronger AKH-C′ immunoreactive band at approximately 42 kDa was detected in AKH+/+ heart-derived plasma rather than in the tail-derived plasma. These findings indicate that AKH-associated immunoreactivity is detectable in circulation, with the major band appearing at approximately 42 kDa (Figure 1C). Densitometric analysis, normalized to GAPDH, confirmed that AKH immunoreactivity was markedly higher in AKH+/+ heart-derived plasma than in AKH-/- heart plasma (9.81 ± 0.22 in AKH+/+ heart versus 0.19 ± 0.03 in AKH-/- heart; *p* = 0.0005), whereas the difference between AKH+/+ tail and AKH-/- tail plasma was not statistically significant (2.71 ± 1.72 in AKH+/+ versus 0.54 ± 0.43 in AKH-/-; *p* = 0.346; Figure 1D).

Together, these results show that AKH immunoreactivity is detectable in plasma and enriched around vascular structures, supporting an association of AKH with the vascular microenvironment.

### 3.2. AKH Deficiency Causes Prolonged Bleeding and Increased Estimated Blood Loss Following Tail Injury

**Figure 2:**
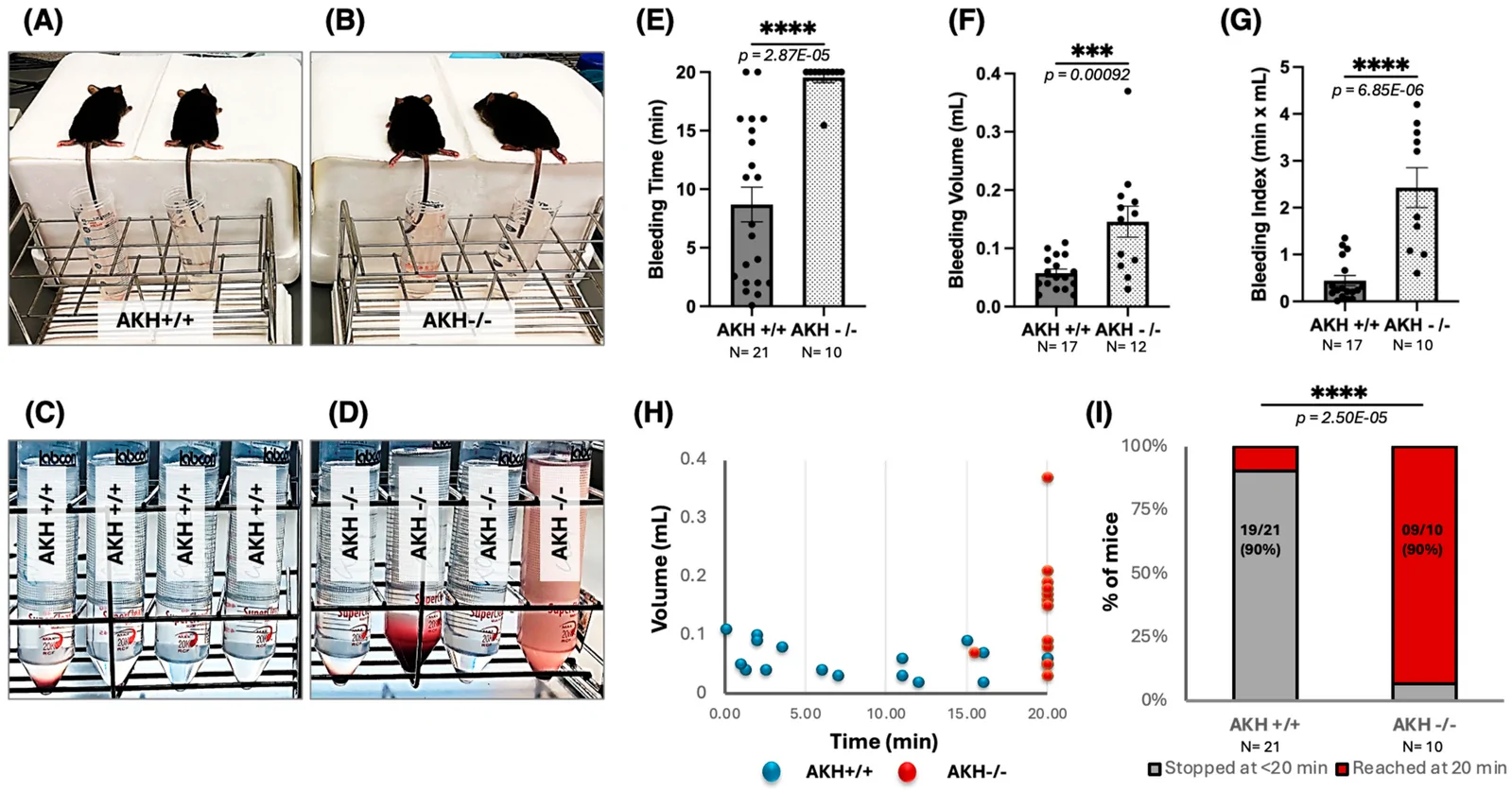
AKH-deficient mice exhibit prolonged bleeding and increased estimated blood loss in the tail bleeding assay. (**A**,**B**) Representative images showing the tail bleeding assay setup for AKH+/+ mice and AKH-/- mice. A distal segment of the tail was amputated, and the tail was immersed in pre-warmed PBS during the observation period. (**C**,**D**) Representative images of blood collection in the PBS containing tubes after the tail bleeding assay in AKH+/+ and AKH-/- mice, showing visibly greater estimated blood loss in AKH-deficient animals. (**E**) Quantification of bleeding time. AKH-/- mice showed a significant prolongation of bleeding time compared with AKH+/+ controls. (**F**) Quantification of bleeding volume. Estimated blood loss was significantly increased in the AKH-/- mice. (**G**) Bleeding index, calculated from bleeding time and bleeding volume, was significantly elevated in AKH-/- mice. (**H**) Scatter plot showing the relationship between bleeding time and bleeding volume in individual animals. (**I**) Percentage of mice in which bleeding stopped before 20 min or continued until the 20 min experimental endpoint. Most AKH+/+ mice stopped bleeding before 20 min, whereas most AKH-/- mice reached the 20 min endpoint. Data are presented as mean ± SEM. Sample numbers are indicated in each panel. Statistical significance was determined as indicated in the figure. For panel I, Fisher’s exact test, two-tailed, was used. *** *p* < 0.001; **** *p* < 0.0001.

To determine whether AKH is required for normal hemostatic control in vivo, we performed the tail bleeding assay using approximately 2-month-old control AKH+/+ and AKH-/- littermates. Following distal tail transection and immersion in pre-warmed PBS, AKH-/- mice showed a robust hemostatic defect, with visibly greater blood accumulation in the collection tubes compared with AKH+/+ controls (Figure 2A–D).

Quantitative analysis confirmed that loss of AKH markedly prolonged bleeding after injury. Mean bleeding time increased from 8.69 ± 1.48 min in AKH+/+ mice to 19.70 ± 0.46 min in AKH-/- mice (*p* = 2.87 × 10^−5^; Figure 2E), indicating that most AKH-deficient animals reached the 20 min experimental endpoint. Consistent with this impaired bleeding phenotype, the estimated blood loss was also significantly elevated in the AKH-/- mice (0.146 ± 0.027 mL) relative to AKH+/+ controls (0.0576 ± 0.0068 mL; *p* = 0.00092; Figure 2F).

Because the bleeding phenotype reflects both bleeding duration and the amount of blood lost, we integrated these parameters into a composite bleeding index. AKH-/- mice exhibited an approximately 5.5-fold increase in bleeding index compared with AKH+/+ mice (2.428 ± 0.424 versus 0.444 ± 0.107 min × mL; *p* = 6.85 × 10^−6^; Figure 2G). Individual animal analysis further showed that the AKH-deficient cohort clustered toward prolonged bleeding time and greater estimated blood loss, whereas most control animals stopped bleeding with comparatively limited estimated blood loss (Figure 2H). A high proportion of AKH+/+ mice stopped bleeding before 20 min, whereas AKH-/- mice were strongly enriched among animals that continued bleeding until the endpoint (Fisher’s exact test, *p* = 2.5 × 10^−5^; Figure 2I). Together, these data identify AKH as a critical regulator of injury-induced hemostasis and show that AKH deficiency produces a pronounced bleeding phenotype characterized by delayed bleeding arrest and increased estimated blood loss.

### 3.3. Healthy Donor Bone Marrow Transplantation Does Not Rescue the AKH-/- Bleeding Phenotype

**Figure 3:**
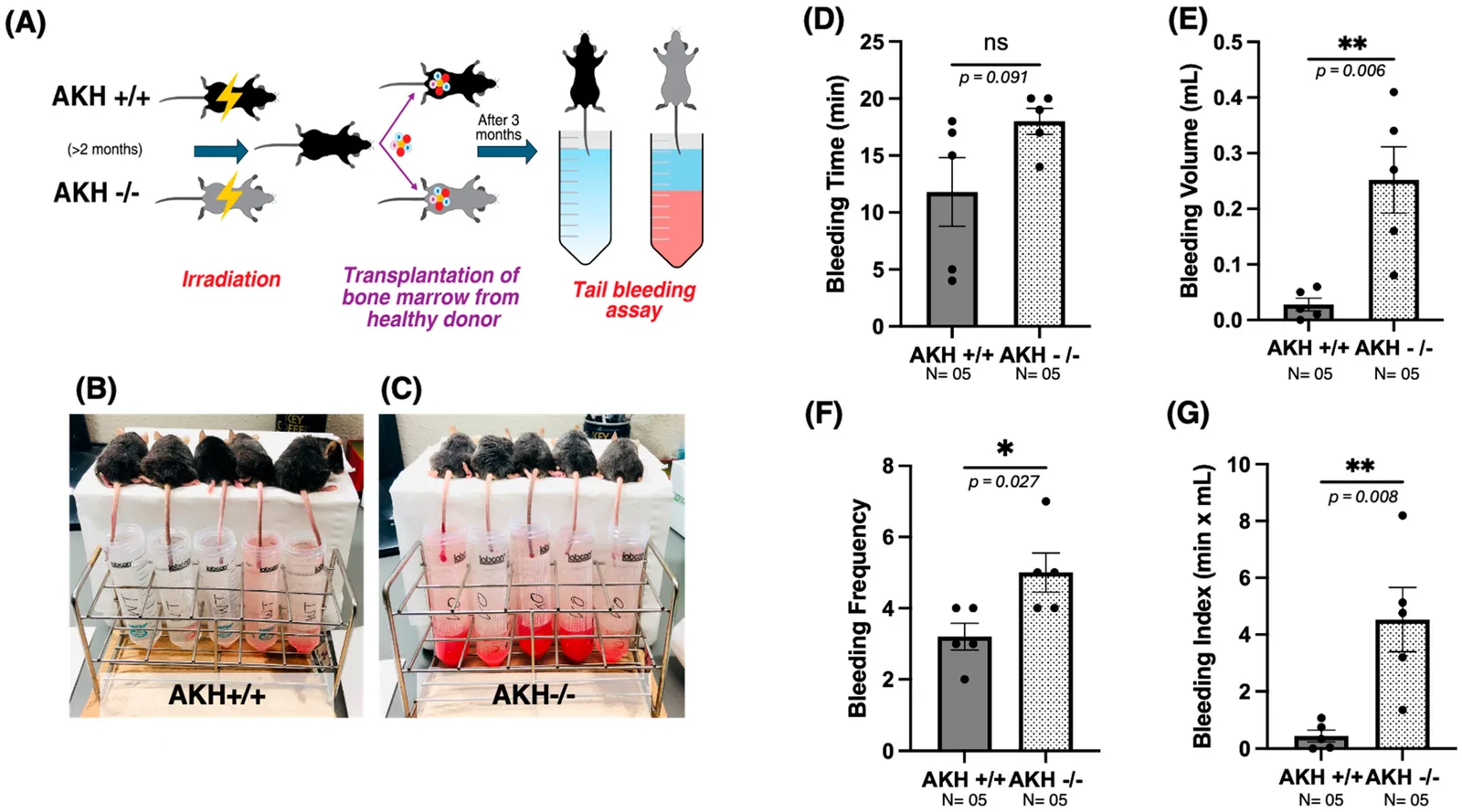
The prolonged bleeding phenotype of AKH-deficient mice is not rescued by transplantation of healthy donor bone marrow. (**A**) Experimental design of the bone marrow transplantation assay. AKH+/+ and AKH-/- recipient 2-month-old mice were irradiated and transplanted with bone marrow from healthy donor mice. After a recovery period of 3 months, mice were subjected to the tail bleeding assay. (**B**,**C**) Representative images of tail bleeding assay from transplanted AKH+/+ and AKH-/- recipient mice. Increased blood accumulation was observed in samples from AKH-/- recipients after transplantation. (**D**) Bleeding time after bone marrow transplantation. Bleeding time remained longer in AKH-/- recipients than in AKH+/+ counterparts, although the difference did not reach statistical significance. (**E**) Bleeding volume was significantly increased in AKH-/- recipients compared with AKH+/+ recipients after transplantation. (**F**) Bleeding frequency was significantly increased in AKH-/- recipients. (**G**) Bleeding index remained significantly elevated in AKH-/- recipients after transplantation. These results indicate that transplantation of healthy donor bone marrow does not normalize the bleeding phenotype of AKH-/- mice, suggesting a contribution from recipient tissue or other non-hematopoietic mechanisms. Data are presented as mean ± SEM. *n* = 5 mice per group. Statistical significance was determined as indicated in the figure. n.s., not significant; * *p* < 0.05, ** *p* < 0.01.

We next asked whether the bleeding phenotype in AKH-deficient mice could be corrected by reconstitution of the hematopoietic compartment. Approximately 2-month-old AKH+/+ and AKH-/- recipient mice were irradiated and transplanted with bone marrow from healthy donor mice. After approximately 3 months of recovery, recipients were subjected to the same tail bleeding assay (Figure 3A). Following WT bone marrow transplantation, AKH-/- recipients continued to show greater blood accumulation than AKH+/+ recipients (Figure 3B,C).

Bleeding time remained elevated in transplanted AKH-/- recipients compared with transplanted AKH+/+ counterparts (18.0 ± 1.14 min versus 11.8 ± 3.02 min), although this difference did not reach statistical significance in this cohort (*p* = 0.091; Figure 3D). In contrast, estimated blood loss was significantly increased after transplantation, with AKH-/- recipients losing approximately nine-fold more blood than AKH+/+ recipients (0.252 ± 0.0596 mL versus 0.028 ± 0.0116 mL; *p* = 0.006; Figure 3E).

The persistence of the phenotype was further supported by bleeding frequency and bleeding index. AKH-/- recipients showed increased bleeding frequency relative to AKH+/+ recipients (5.0 ± 0.55 versus 3.2 ± 0.37; *p* = 0.027; Figure 3F), and the bleeding index remained markedly elevated (4.530 ± 1.134 versus 0.442 ± 0.208 min × mL; *p* = 0.008; Figure 3G). Thus, transplantation of healthy donor bone marrow was insufficient to normalize hemostasis in AKH-deficient recipients. Thus, WT bone marrow transplantation did not normalize hemostasis in AKH-deficient recipients, suggesting that hematopoietic replacement alone does not account for the AKH-dependent effect.

### 3.4. AKH Deficiency Shortens APTT Without Affecting PT

**Figure 4:**
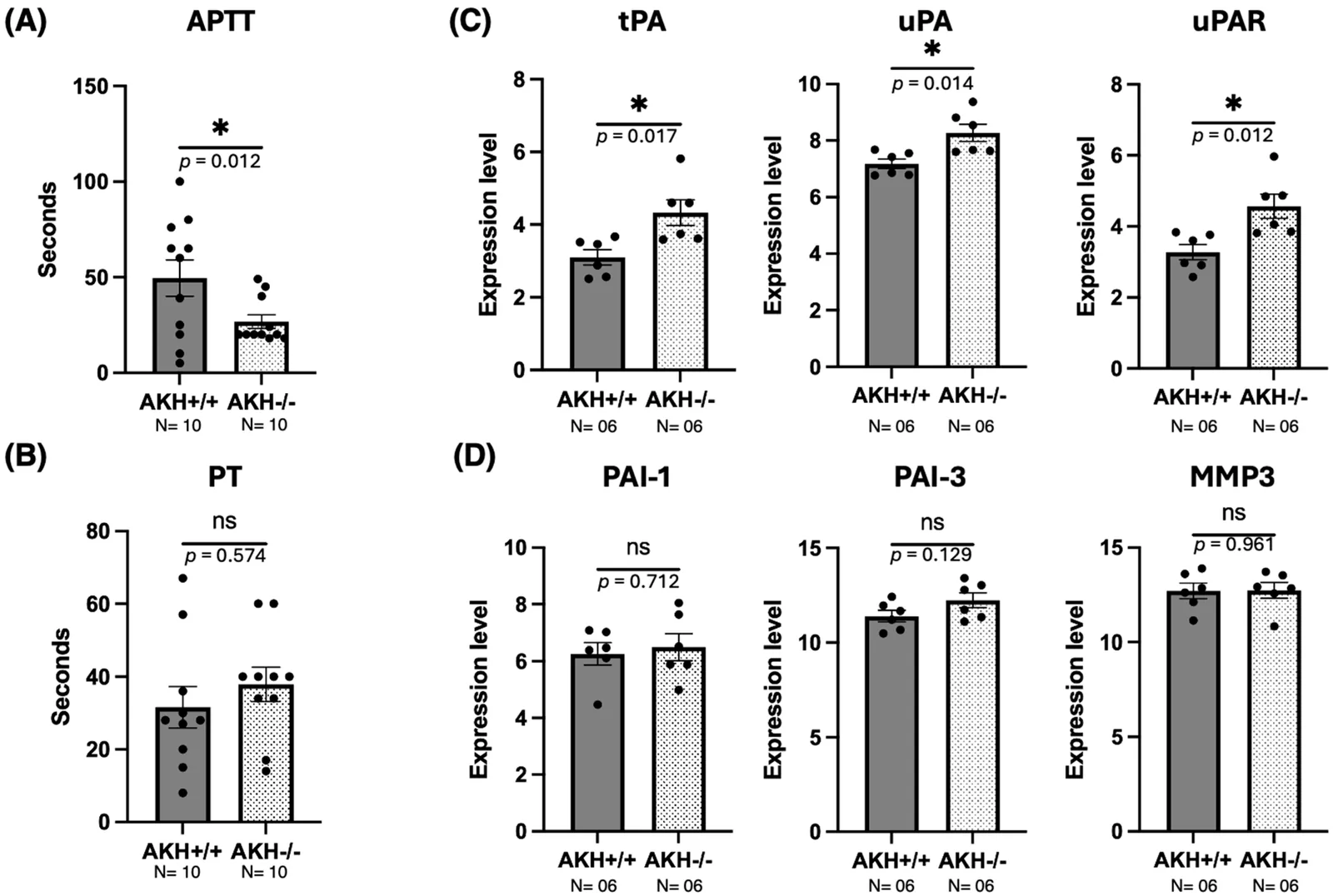
Blood coagulation activity and qPCR analysis of fibrinolysis-related transcript levels in plasma from AKH+/+ and AKH-/- animals. (**A**) Activated partial thromboplastin time (APTT) was measured in plasma from AKH+/+ and AKH-/- animals. AKH-/- animals showed a significantly shorter APTT than AKH+/+ animals. (**B**) Prothrombin time (PT) was measured in plasma from AKH+/+ and AKH-/- animals. PT did not differ significantly between genotypes. (**C**) Transcript levels of tissue-type plasminogen activator (tPA), urokinase-type plasminogen activator (uPA) and urokinase-type plasminogen activator receptor (uPAR) were significantly elevated in AKH-/- mice. (**D**) Transcript levels of plasminogen activator inhibitor-1 (PAI-1), plasminogen activator inhibitor-3 (PAI-3), and matrix metalloproteinase-3 (MMP-3) were not significantly altered between genotypes. Data are shown as mean ± SEM with individual data points overlaid. Statistical significance is indicated by the *p* values shown above the graphs. n.s., not significant; * *p* < 0.05.

In the preceding experiments, AKH-/- mice showed prolonged bleeding time (Figure 2), and bone marrow transplantation did not rescue this bleeding phenotype (Figure 3), suggesting that the hemostatic defect may arise from the peripheral tissue microenvironment, including the vascular compartment, rather than from bone marrow-derived cells. To further determine whether AKH deficiency affects the classical plasma coagulation system, we measured APTT and PT in plasma derived from adult AKH+/+ and AKH-/- mice. APTT was significantly shortened in AKH-/- mice compared with AKH+/+ controls (49.5 ± 9.48 s in AKH+/+ controls versus 26.73 ± 10.13 s in AKH-/- mice; *p* = 0.012; Figure 4A). In contrast, PT was not significantly different between AKH+/+ and AKH-/- mice (31.6 ± 6.3 s in AKH+/+ controls versus 37.9 ± 7.42 s in AKH-/- mice; *p* = 0.574; Figure 4B).

Next, we examined whether AKH deficiency affects the fibrinolytic system by measuring transcript levels of fibrinolysis-related genes in plasma samples from adult AKH+/+ and AKH-/- mice. Transcript levels of tPA (3.10 ± 0.315 in AKH+/+ versus 4.327 ± 0.358 in AKH-/- mice; *p* = 0.017), uPA (7.179 ± 0.270 in AKH+/+ versus 8.274 ± 0.305 in AKH-/-; *p* = 0.014), and uPAR (3.278 ± 0.326 in AKH+/+ versus 4.569 ± 0.381 in AKH-/-; *p*= 0.012) were significantly increased in AKH-/- mice compared with AKH+/+ controls (Figure 4C). In contrast, the expression levels of plasminogen activator inhibitor-1 (PAI-1, 6.257 ± 0.390 in AKH+/+ versus 6.492 ± 0.478 in AKH-/- mice; *p* = 0.712) and plasminogen activator inhibitor-3 (PAI-3, 11.398 ± 0.309 in AKH+/+ versus 12.233 ± 0.392 in AKH-/- mice; *p* = 0.129), which inhibit plasminogen activator activity, were not significantly altered between genotypes (Figure 4D). Matrix metalloproteinase-3 (MMP-3) transcript levels were also unchanged in AKH-/- mice (12.708 ± 0.409 in AKH+/+ versus 12.737 ± 0.442 in AKH-/- mice; *p* = 0.961; Figure 4D).

These screening assays indicate that AKH deficiency is associated with a selective shortening of APTT without a detectable change in PT, suggesting that AKH may influence specific aspects of the coagulation system. At the same time, the fibrinolysis-related transcript profile showed selective upregulation of key activators rather than broad changes in fibrinolytic or extracellular matrix-remodeling genes. Together, these findings provide a possible context for the abnormal tail-bleeding phenotype that is not explained by WT bone marrow transplantation or the shortened APTT.

### 3.5. Wound Repair Delays in the Absence of AKH

**Figure 5:**
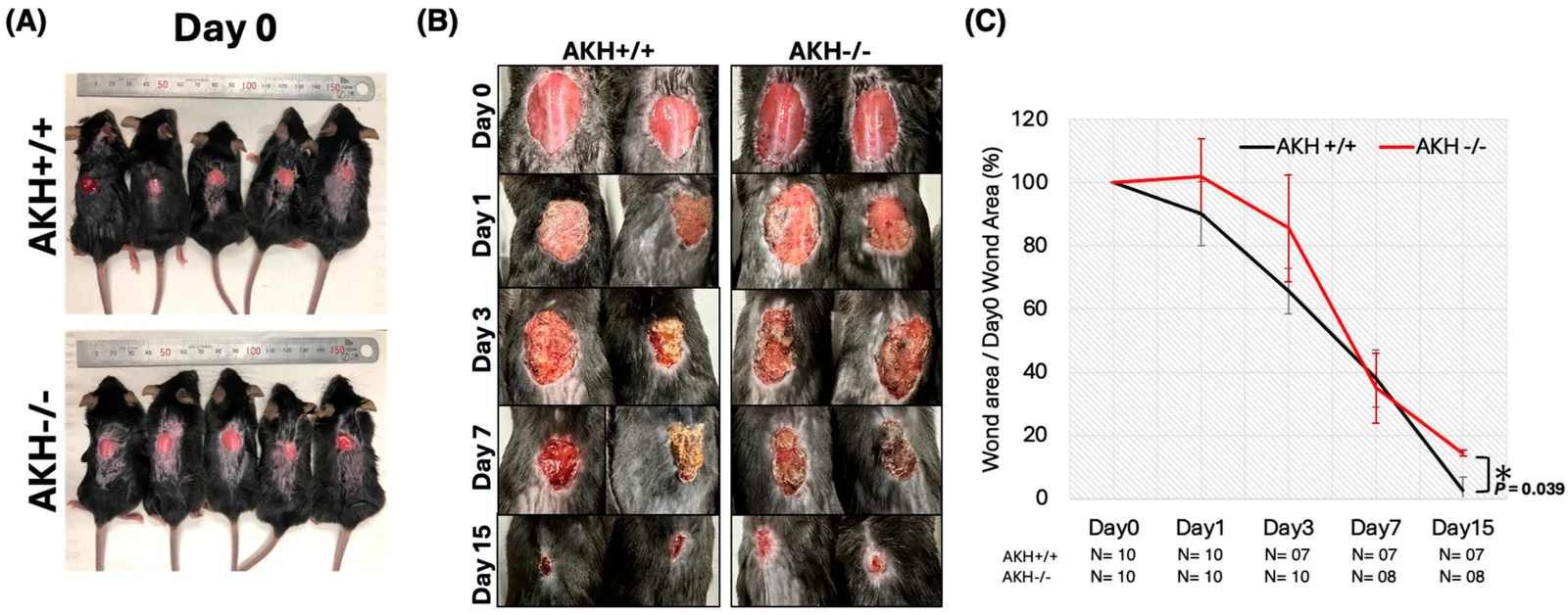
AKH deficiency induces delayed wound closure after skin injury. (**A**) Representative images of AKH+/+ and AKH-/- mice immediately after wound introduction at Day 0. (**B**) Representative serial images of wound closure in AKH+/+ and AKH-/- mice at Day 0, 1, 3, 7, and 15. (**C**) Quantification of wound area over time. Wound areas were comparable between genotypes during the early phase, whereas AKH-/- mice retained a significantly larger residual wound area at Day 15. Data are shown as mean ± SEM; *n* = 10 mice per genotype at baseline. Differences in residual wound area between genotypes at Day 15 were analyzed using an unpaired two-tailed Welch’s *t*-test. Statistical significance is indicated in the figure. * *p* < 0.05.

We next examined wound-healing dynamics in the absence of AKH, because the hemostatic clot serves as a provisional matrix for tissue repair. Comparable injuries were introduced, with similar initial wound sizes between AKH+/+ and AKH-/- mice on Day 0 (Figure 5A). Serial images acquired on Days 0, 1, 3, 7 and 15 documented wound closure, with AKH-/- mice showing greater persistence of residual wound area, particularly during the later phase of healing (Figure 5B). Quantitative analysis showed that wound area decreased over time in both genotypes. Although wound size was not consistently larger in AKH-/- mice during the early phase of healing, AKH-/- mice retained a significantly larger residual wound area by Day 15 compared with AKH+/+ mice (2.79 ± 3.56% in AKH+/+ mice versus 14.95 ± 1.15% in AKH-/- mice; *p* = 0.039; Figure 5C). These data demonstrate that AKH deficiency is associated with delayed late-stage wound resolution. Together with the prolonged bleeding phenotype and altered expression of fibrinolysis-related genes, this result suggests that AKH may contribute not only to bleeding control but also to subsequent tissue repair.

## 4. Discussion

Our investigation demonstrates that AKH acts as a non-hematopoietic molecular cue that contributes to hemostasis and wound repair in mice. The findings support a model in which the absence of AKH leads to excessive bleeding and delayed wound closure without showing a typical defect in the classical coagulation cascade. Rather, AKH appears to contribute to the stability and repair competence of the injured tissue interface, thereby extending its role from hemorrhage control to wound healing.

Previous studies have established that AKH functions as an ECM cue containing two von Willebrand factor A domains and one LCCL domain and exhibits heterophilic cell adhesion properties [15,16]. To date, the published literature has described AKH almost exclusively in the developmental and neural context [17,18,19,22]. AKH has been shown to regulate the proliferation and differentiation of neural stem/progenitor cells in the spinal cord as well as in the major neurogenic niches of mouse brain [17,18]. Recently, AKH has also been implicated in innate immune barrier function within the neurogenic niches [19]. All these studies point to a recurring theme that AKH functions at specialized extracellular interfaces where structural organization, local signaling, and tissue protection must be tightly regulated.

In this study, we identify a function of AKH outside the nervous system, broadening its potential role to vascular injury, hemostatic stability and tissue repair. The core strength of this study is the physiological coherence of the phenotype. Our observation of AKH enrichment around arterial structures, together with the excessive bleeding phenotype in AKH-deficient mice, supports an association between AKH and hemostatic wound repair. These two processes are tightly regulated through crosstalk among the regulatory cues in the vascular microenvironment [23,24,25,26], positioning AKH as a candidate for non-hematopoietic and tissue-derived regulator. This interpretation is supported by the bone marrow transplantation experiment, in which WT donor bone marrow did not rescue the bleeding phenotype of AKH-deficient recipients, consistent with a predominant non-hematopoietic or tissue-resident contribution. The hemostatic clot not only acts as a first line of defense to limit blood loss at the injury site but also serves as a provisional matrix that supports inflammatory infiltration, stromal activation, angiogenesis and tissue reconstruction [27,28]. Factors that destabilize clot persistence or clot remodeling can increase bleeding and impaired repair [7,13,29]. Therefore, our analysis places AKH within the wider arena of vascular injury biology, rather than as an isolated hematologic observation.

Interestingly, the coagulation assays revealed an apparent paradox in relation to the bleeding phenotype [30,31]. PT was unchanged, whereas APTT was shortened in the absence of AKH. These do not suggest a simple defect in the classical coagulation cascade [1,2]. Instead, they suggest that the dominant effect may lie in the local organization, stabilization or remodeling of the hemostatic plug after injury rather than in defective initiation of clot formation per se [24,29,32,33]. This interpretation is further supported by the increased expression of tPA, uPA, and uPAR in AKH-deficient samples. Fibrin turnover and extracellular proteolysis are highly coordinated by the plasminogen activator system [8,9,20,34]. However, this system extends beyond fibrinolysis and regulates inflammatory cell migration, matrix remodeling, angiogenesis and wound healing [10,35,36,37,38,39]. Our results provide a pivotal molecular pattern consistent with the in vivo phenotype and support a model in which loss of AKH disrupts the balance between clot preservation and clot resolution [11,12,40]. This framework may help explain why bleeding is enhanced while wound closure is delayed, since both processes depend on maintaining the provisional matrix for an appropriate duration [4,27,28].

The wound-healing phenotype further supports a role for AKH not only in bleeding control but also in the broader reparative program that follows injury [3,41]. These findings highlight how extracellular, non-hematopoietic regulators in the local tissue environment can influence two distinct and clinically important outcomes: susceptibility to bleeding and efficiency of healing [25,26,42]. Thus, AKH demonstrates a unique category of injury response regulatory cue that bridges vascular stability to regenerative competence.

Further investigations will be required to address several important questions raised by this study. The mechanistic model remains inferential, as direct analyses of fibrin architecture, plasmin generation, clot-lysis kinetics, platelet function or endothelial signaling have not yet been performed [29,32,40]. In addition, increased expression of tPA, uPA and uPAR is insufficient to confirm that enhanced fibrinolysis is the primary driver of the phenotypes [11,14,34]. Furthermore, the exact source of AKH and the immediate target cells or matrices through which it acts in the vascular injury microenvironment remain to be determined [25,26,43]. A direct immunohistochemical analysis of injured tail tissue, including fibrin/platelet deposition and vascular or perivascular matrix markers, will be important for testing whether AKH contributes to the clot–wound interface. It will also be important to determine whether AKH deficiency secondarily alters VWF expression or activity, platelet function or platelet–endothelial signaling interactions.

**Figure 6:**
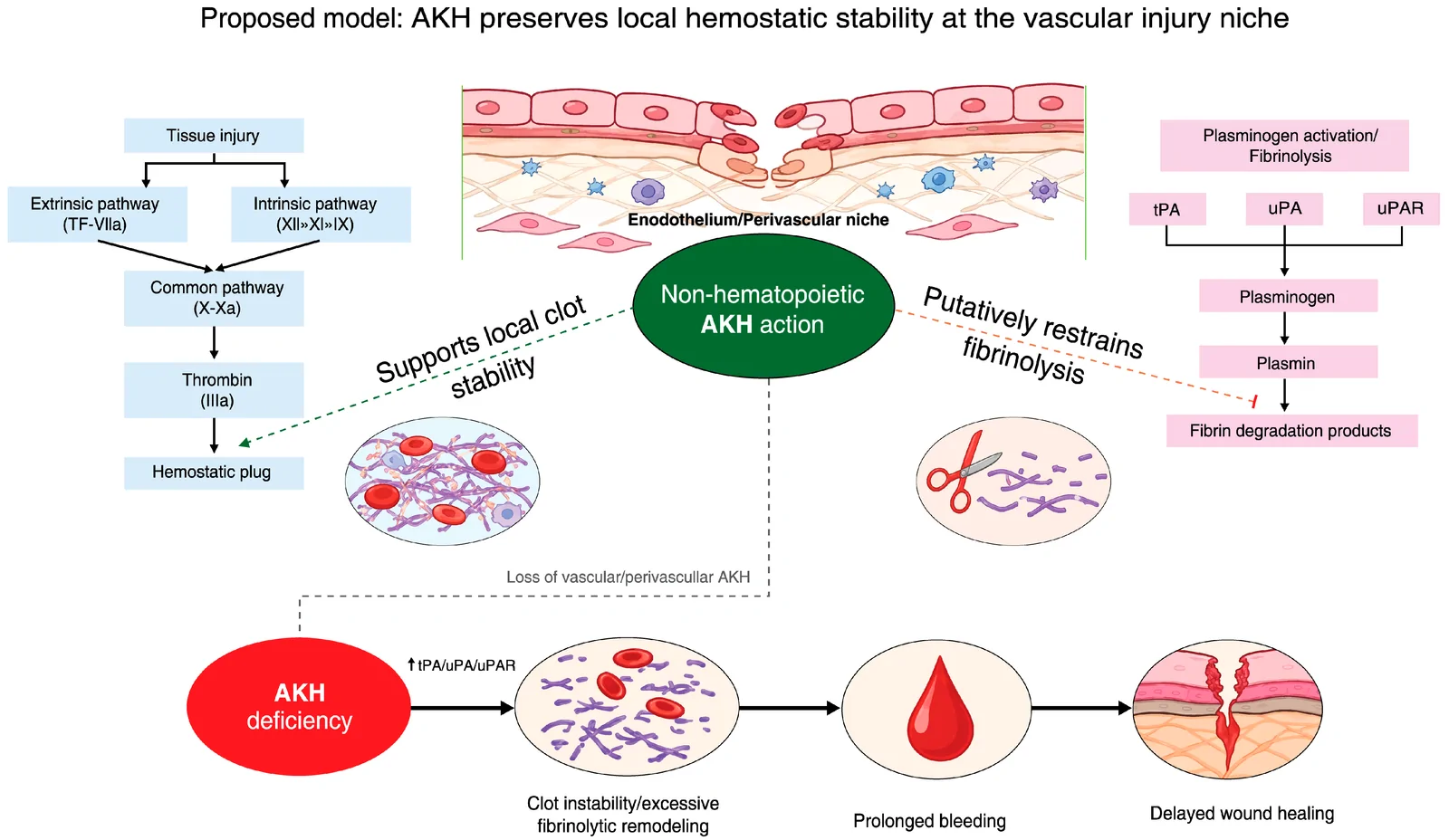
Proposed model of AKH function in hemostatic wound repair. AKH is proposed to act within the vascular/perivascular injury niche to preserve hemostatic stability. Following vascular injury, activation of the classical coagulation cascade leads to thrombin generation and the formation of a fibrin-rich hemostatic plug. AKH plausibly supports local clot stability while putatively restraining plasminogen-associated fibrinolysis. In AKH deficiency, increased tPA/uPA/uPAR expression is associated with prolonged bleeding and delayed wound closure; altered fibrinolytic remodeling and clot stability are proposed components of the working model. Dashed arrows indicate proposed AKH-dependent effects and inhibitory bar indicates putative restraint of fibrinolysis.

Based on our combined evidence, we propose a working model in which AKH acts locally within the vascular/perivascular niche to maintain hemostatic stability (Figure 6). In this model, AKH is proposed to support local clot stability after a vascular injury and may putatively restrain plasminogen activation associated fibrinolytic remodeling. Loss of AKH in the vascular/perivascular niche may therefore shift the injury microenvironment toward increased tPA/uPA/uPAR related remodeling, potentially resulting in clot instability, prolonged bleeding and delayed wound healing. Although this model remains conjectural, it provides a coherent framework linking the proposed non-hematopoietic action of AKH with the observed abnormalities in bleeding, fibrinolytic regulation and efficient wound repair.

## 5. Conclusions

In conclusion, AKH deficiency is associated with prolonged bleeding, increased estimated blood loss and delayed late-stage wound closure. WT bone marrow transplantation did not normalize the bleeding phenotype, suggesting that hematopoietic replacement alone is insufficient to restore normal hemostatic control. Our investigation ultimately identifies AKH as a non-hematopoietic and ECM-associated regulatory cue in peripheral biology, extending its biological relevance beyond neural niches.

## Data Availability

The original data presented in this study are openly available upon request to corresponding authors.

## Abbreviations

AKH	Akhirin
APTT	activated partial thromboplastin time
BM	bone marrow
BMT	bone marrow transplantation
BSA	bovine serum albumin
DMEM	Dulbecco’s modified Eagle’s medium
ECM	extracellular matrix
EDTA	ethylenediaminetetraacetic acid
GAPDH	glyceraldehyde-3-phosphate dehydrogenase
IHC	immunohistochemistry
LCCL	Limulus factor C, Coch-5b2, and Lgl1 domain
MMP	matrix metalloproteinase
NaCl	sodium chloride
OCT	optimal cutting temperature compound;
PAI	plasminogen activator inhibitor
PFA	paraformaldehyde
PBS	phosphate-buffered saline
PT	prothrombin time
PVDF	polyvinylidene difluoride
qPCR	quantitative polymerase chain reaction
SDS	sodium dodecyl sulfate
TBS-T	Tris-buffered saline with Tween 20
tPA	tissue-type plasminogen activator
Tris-HCl	tris (hydroxymethyl)aminomethane hydrochloride
uPA	urokinase-type plasminogen activator
uPAR	urokinase-type plasminogen activator receptor
